# The Prevalence of Cataract in Children

**DOI:** 10.7759/cureus.30135

**Published:** 2022-10-10

**Authors:** Dishika Katre, Kanchan Selukar

**Affiliations:** 1 Ophthalmology, Jawaharlal Nehru Medical College, Datta Meghe Institute of Medical Science, Wardha, IND

**Keywords:** etiology, red reflex, leukocoria, uveitis, opacification, genetics, epidemics

## Abstract

Cataract is the leading cause of childhood blindness in developing countries. Early detection and treatment of childhood cataracts can reduce the burden of blindness in the nation. Often the etiology of pediatric cataract is idiopathic; however, genetics play a role in the development of congenital cataract. According to epidemiologists, one-fourth of cases of congenital cataracts are hereditary. Gene responsible for the development of cataract is identified using gene mapping, which helps to prevent future blindness in the family. Cataracts can also present with systemic disease, microphthalmia, microcornea, and aniridia. The presentation of cataracts varies in individuals, some are symptomatic while others are asymptomatic. Parents after noticing strabismus and leukocoria bring their children to an ophthalmologist. Early diagnosis can restore visual function in cases of congenital cataract. In young babies, the type of cataract is determined using slit-lamp examination and examination under anesthesia in OR. Most cases of pediatric cataracts are accidental findings during routine checkups. On direct ophthalmoscopy, red reflex is not appreciated in cases of cataracts. Advancing technology changes the ophthalmologist's approach to pediatric cataract surgery, improving postoperative refractory function. In children, minor incision surgery was preferred to heal early. An appropriate choice of intraocular lens (IOL) should be made for implantation in a child's eye to avoid postimplanted complications. Inflammation and amblyopia affect the outcome of treatment. Complications of cataract surgery include posterior capsule opacification, glaucoma, inflammation, and uveitis.

## Introduction and background

The opacity of the crystalline lens is called a cataract. Childhood cataract is rare compared to age-related senile cataract. Any opacity found within two months of birth is said to be a congenital cataract, and opacity spotted in infants and early childhood is referred to as developmental cataract [1]. Congenital cataract causes an irreversible visual field defect caused by visual deprivation during the sensitive period of visual development [2]. The standard types of cataracts found in young infants are congenital and developmental cataracts that occur due to disturbance in the normal development process of the eye. If it is a congenital cataract, opacities are present in the embryonic and fetal nucleus. Onset of developing cataracts ranges from infant to teenager [3,4]. Unilateral cataract in children leads to amblyopia in the other eye. Congenital cataract, also known as neonatal cataract, occurs in the first year of life. The onset of juvenile cataract occurs within the first decade of life [5]. Other leading causes of blindness in children that are treatable include vitamin A deficiency, measles, toxoplasmosis, others (syphilis, hepatitis B), rubella, cytomegalovirus, herpes simplex virus (TORCH), and cataract [6]. In 1992, WHO came up with a program - Vision 2020: The Right to Sight; the objective of this program was to eliminate blindness worldwide by 2020 [7].

## Review

Epidemiology

Childhood blindness can be reduced by early diagnosis and prompt treatment. Childhood cataracts are the second most common cause of blindness in the world after adult-onset cataract. In Asia, the incidence of childhood blindness is more than one million, and the most common cause of this is pediatric cataract [6]. A developing nation like India contributes 7.4-15.3% of the total incidence of blindness, which reduces those children's quality-adjusted life years. The prevalence of childhood cataracts is higher in developing countries due to the low standard of living; the incidence of childhood cataracts remains the same irrespective of sex and education status in the community. A study showed that 67% of pregnant mothers of children with congenital cataract suffered from illness, and 22% of expectant mothers were on medication. About 22% of congenital cataracts present with a systemic disorder, and 27% of congenital cataracts have ocular dysfunction. Diagnosis of cataract in children is an accidental finding during routine eye checkups [4,8].

Genetic/molecular

At 22 days of gestation, the eye starts developing and the lens develops from the surface ectoderm. Most fibroblast growth factors produced in vitreous are essential for the differentiation of secondary lens fiber as the polarity of the lens is due to fibroblast regulating growth factor (FGF) [9]. PAX6, PITX3, c-Maf, and FOXE3 are genes that code for proteins that play the role of transcription factor in the development of the lens. Mutation of any protein results in a defective production of the lens. The anterior epithelial cells of lens preserve their morphology and proliferative ability while posterior epithelial cells form primary lens fiber [10]. Some pediatric cataracts have a family history. Most congenital cataracts occur due to a sporadic mutation in a gene leading to autosomal dominance. Cataract phenotype variation results from a mutation in CRYAA, CRYAB, CRYBB1, CRYBB2, CRBB3, CRYGC, and CRYGD genes; the alpha, beta, and gamma genes are responsible for maintaining the transparency of the lens [11]. Nuclear, lamellar, zonular, and posterior pole cataracts occur in alpha gene mutation [12]. Connexin gene protein forms an integral part of lens gap junction through connexin gene exchange. Mutation in the connexin50 gene leads to a loss of lens transparency [13]. Congenital nuclear cataract has autosomal dominant inheritance [14].

Etiology

Idiopathic

Most unilateral and bilateral cataracts are of unknown etiology. They are confirmed after ruling out all causes [3].

*Hereditary* *Cataract and Down Syndrome*

Congenital cataracts are autosomal dominant hereditary cataracts with incomplete inheritance, as they have not been associated with any syndrome but with ocular defects [3]. A cataract is a common presentation in a child with Down syndrome, with an incidence of 6-50%. Other ocular abnormalities include strabismus, nystagmus, and inward and outward eyelid folding [15].

*Lowe* *Syndrome*

It is identifiable at birth due to ocular manifestation and decreased muscle tone. It is an X-linked multisystem disorder characterized by the triad of congenital cataracts, intellectual disability, and proximal renal tubular dysfunction. Their life expectancy is around 40 years [14,16].

*Juvenile* *Arthritis*

Juvenile chronic arthritis with antinuclear antibodies involvement patients are at risk of developing secondary cataracts with uveitis. Uveitis can be anterior, posterior, intermediate, and panuveitis. Uveitis may or may not be associated with systemic disease. Commonly anterior uveitis cases are reported [3].

Metabolic cataract - galactosemia

The galactose level is abnormally increased in the serum due to mutations in the enzyme galactokinase (GALK1), galactose-1-phosphate uridyltransferase, or uridine diphosphate 1-4-epimerase; this causes osmatic damage to the lens leading to "oil droplet cataract" [16]. Its presentation is as a nuclear cataract and sometimes as anterior and posterior subscapular cataract. It can be reversed by removing galactose from the diet. It is also associated with vomiting, failure to thrive, jaundice, and mental retardation [17].

TORCH

Toxoplasmosis, rubella, cytomegalovirus, herpes, and syphilis congenital infection are associated with congenital cataract. Incidences of congenital cataracts are more familiar with rubella infection. Other ocular manifestations include pigmentary retinopathy, microphthalmos, glaucoma, iris dystrophy, or chorioretinitis. The incidence of toxoplasmosis, others (syphilis, hepatitis B), rubella, cytomegalovirus, herpes simplex virus (TORCH) is more commonly found in developing countries such as India. This group of pathogens affects lens development by acting on the surface ectoderm. The impact of TORCH infection depends on the gestational age at which the mother acquired the infection. In response to the infection, IgM antibodies are raised in the serum of the fetus. IgM can be detected by fetal tissue biopsy [18].

Persistent hyperplastic primary vitreous

It is a congenital anomaly present at birth due to failure of the hyaloid artery and primary vitreous to subside during embryogenesis. It is due to anterior segment involvement and usually affects one eye with no other systemic abnormalities. It usually presents with anterior and posterior segment involvement in the same eye. This occurs due to persistence of fetal fibrovascular lens fiber at birth, with an increased risk of developing secondary glaucoma and secondary hemorrhage [19].

Traumatic cataract

Traumatic cataracts have different etiology in developing and developed countries. It varies from a wooden stick, thorn, bullhorn, or bite of an animal in developing countries to a firecracker injury in a developed country. The incidence of traumatic cataracts is reported more in a boy child than in a girl child. Based on the object causing trauma, they are categorized as blunt and sharp trauma, based on globe involvement further classified as open and close globe injury. In this case, cataracts occur due to shallowing of the anterior chamber, iris distortion, corneal tear, bleeding, and leakage of the vitreous. Management of traumatic cataracts depends on the amount of ocular disturbance and the stage at which the child is present in hospitals [20]. Intracapsular cataract surgery is preferred for subluxation of lens occurring due to injury [21,22].

History taking

**Figure 1:**
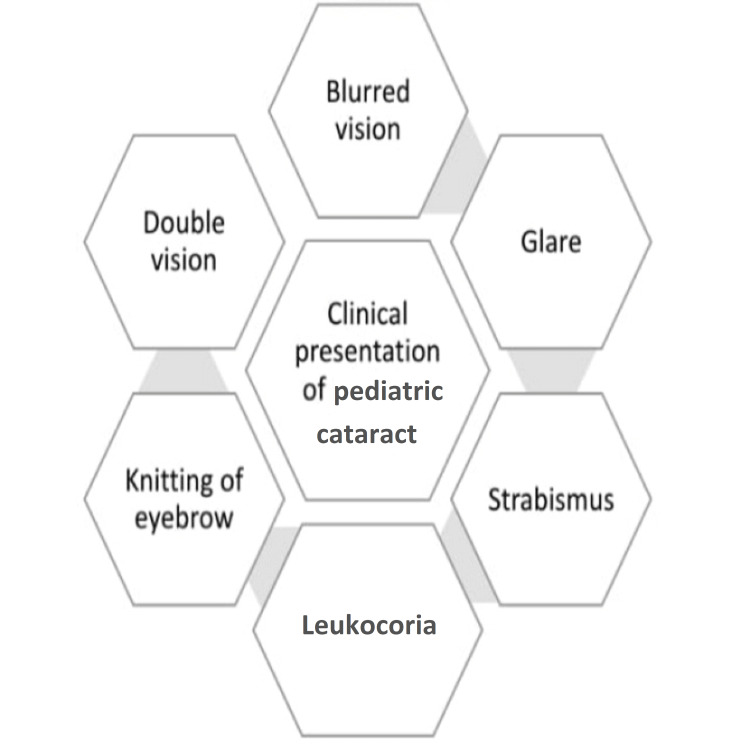
Clinical presentation in pediatric cataract. The image is created by the authors of this study.

Leukocoria, which the parent observes as a total or progressive increase in opacity size, is frequently the first complaint. The second complaint is a youngster not making eye contact with an object close to their face or maintaining gaze (inability to recognize mother). A parent could also lament the straining and tightening of their eyes in solid light. Huge eyes (buphthalmos), small eyes (microphthalmos), and aberrant eye movements (nystagmus) are seen (Figure 1). History should be taken for the duration of symptoms and age of onset. In case of unilateral cataract history of a TORCH infection, alcohol and drug history should be ruled out. Older children may have trouble seeing far items, the instructor may note the child cannot read the board, or the parent may see the child putting things very close to them like observing television up close and nearby. The same history of a current diagnosis in a family member or sibling pedigree chart should be created, and cataract should be sought [23].

Systemic examination

Many times, cataract is associated with systemic disease. On gross examination of individual ocular abnormalities such as spares eyebrows in Down syndrome are found. Children with cataracts frequently exhibit syndromic symptoms and systemic disease. A general physical examination may reveal dysmorphic characteristics, such as sparse eyebrows, a mongoloid slant (Down syndrome), a beaked nose with dental abnormalities (Hallermann-Streiff syndrome), low-set ears, prominent parietal and occipital eminences; skin and hair changes, such as dry, scaly skin over the limbs, abdomen, and scalp with patchy alopecia; and skeletal modifications [24].

Assessment of visual acuity

Checking visual acuity in children is the art of medicine. It is considered a criterion for measurement of the visual function of a child. Visual acuity in kindergarten children is appreciated through single letter and picture recognition, unlike adults who use the Snellen chart. The child is ruled out for squint and nystagmus during visual acuity. The visual evoked potential (VEP) test is the most commonly used test for getting rapid visual acuity results in children [25]. Teller acuity chart is used for measuring the visual system development; in the infant from a study, it is reported that pre-term infants have poor test results. Visual-evoked potential, optokinetic nystagmus, and visually-evoked cortical potential are preferred methods to test visual acuity in infant and preverbal children. These tests have impaired results if the child has any ocular abnormalities. Visual acuity improves from birth to adolescent age [26].

Medical checkup for cataract

Children with congenital cataracts have poor central and peripheral vision. All newborns are screened at birth for ocular abnormalities by direct ophthalmoscope; red light reflex is appreciated. Any deviation in red reflex is seen as ocular abnormalities present in the child. Children are also screened in the maternity ward for early detection of ocular deformities. In the case of unilateral congenital cataract, early diagnosis prevents the development of amblyopia in affected eye. Screening a child at birth for ocular abnormalities is essential for seeing a disturbance in visual system development. The red reflex is seen in the child without using mydriatic eye drops, and the outcome has 85% sensitivity [25].

Presurgical examinations

Before operating on the child, the child undergoes all ocular examinations and eye checks using a direct ophthalmoscope without dilating the pupil, as the pupil dilates opacity becomes less significant. In children with central opacity in the lens, red reflex is not appreciated on direct ophthalmoscopy. Children with bilateral corneal opacity, nuclear cataract >3 mm, or squint are preferable candidates for surgery. Both eyes of a child should be examined for cataract. The detection rate of monocular cataracts is poor at an early stage of life; most of the time, there is no such family history noted and the baby is healthy [26]. A child with rare X-linked recessive disease such as Nance-hance syndrome presents with lenticular cataract and other teeth anomalies and a heterozygous girl child presents with posterior sutural cataract [27].

Intraocular lens power calculation

As the child grows, the eye also grows; the eye's power changes with the person's age. Due to this reason calculating the power of intraocular lens (IOL) before surgery is important to keep in mind. The axial length of the eye and the cornea's refractive power is used for calculating IOL power. In children, under-correction varies with age group as follows: one to two years by 20%, two to four years by 15%, and four to eight years by 10% [28]. Sanders-Retzlaff-Kraff II (SRK2) formula is used for the calculation of IOL power in younger children [29]. Various factors affecting the power of IOL include the age of the child, type of cataract, visual acuity, congenital or developmental, axial length and power of cornea at presentation, refractive error, and involvement of single or both eye [30]. It has been seen from a study that myopic shift is more in the affected eye compared to a normal eye. Criteria for implantation of IOL - axial length should be >17 mm and corneal diameter >10 mm [31].

Choice of IOL

In the early era, polymethyl methacrylate (PMMA) IOL being cheap was used in India. Now, PMMA IOL is replaced by heparin-surface modified PMMA IOL because it has greater biocompatibility. Nowadays, pediatric ophthalmologists prefer to use foldable acrylic hydrophobic IOL in younger children as it requires a small incision due to its soft nature with few episodes of postoperative inflammation and refractive error (astigmatism). In a child, IOL is implanted in the sulcus. More frequency of capsule contraction is observed in a child with silicon IOL implantation. In a child's small eye, monofocal IOL is chosen over multifocal IOL. With increasing age, the axial length of a child's eye increases to 23.6 mm, which at birth was 16.8 mm, and the refractive power of the cornea declined from 51 diopters at birth to 45 diopters at six months of age and reached 43.5 diopters in adulthood. Refractive error does not remain constant and changes from infancy to adulthood. Hydrophobic IOL is not used because it causes posterior capsule opacification and leads accumulation of calcium deposits on IOL [31]. Bag-in-the-lens implantation technique reduced the incidence of posterior capsular opacification [32].

Surgery

Ophthalmologists prefer closed chamber surgery in the pediatric age group. The child has lower corneal and scleral rigidity, a very elastic anterior capsule, a soft lens, and well-formed vitreous. The superior incision is preferred in the child. Anterior and posterior continuous curvilinear capsulorhexis is performed with a viscocohesive (HEALON GV; Johnson & Johnson Vision: Santa Ana, CA) or viscoadaptive (HEALON 5; Johnson & Johnson Vision: Santa Ana, CA) device. During surgery, chamber stability is maintained using 23.25-gauge scissors and forceps [33]. Preschool children's anterior capsulectomy is performed using a vitrectomy cutter. Superior incision protects the wound by lid and bell's phenomenon. The incision can be scleral or corneal; two side ports are created at 180° apart, giving 360° movement. Precaution should be taken at the time of creating side port to prevent shallowing of the chamber. A pupil is dilated using preservative-free adrenaline (1:100,000), and trypan blue dye (0.06%) under air stain is used to stain capsule. Nick on the anterior capsule is created using cystectomy. Capsulorhexis is done by using Utrata forceps or intravitreal 23-gauge forceps by making a 2.2 mm incision. The desired size of capsulorhexis is achieved through two incisions push-pull technique [25]. Multi-quadrant hydro dissection (>3 quadrants) is done, and lens matter in children is removed through bimanual lens aspiration. The viscoelastic substance partially fills the anterior chamber; the posterior capsule is managed here to avoid visual axis opacification. Nick on the posterior capsule is given with cystotome, and posterior capsulorhexis is performed through intravitreal forceps. Posterior capsulorhexis is essential in all children <six years of age [27].

In a child with subluxation of lens, intravitreal aspiration of lens with anterior chamber IOL and peripheral iridectomy is the treatment of choice. To reduce the risk of anterior chamber collapse and endophthalmitis, surgical incision is sutured using 10-0 monofilament nylon. If no leakage is found, then the side port is left sutureless [34]. In traumatic cataracts, to avoid visual axis opacification, posterior capsulorhexis is done [35].

Complication of surgery

**Figure 2:**
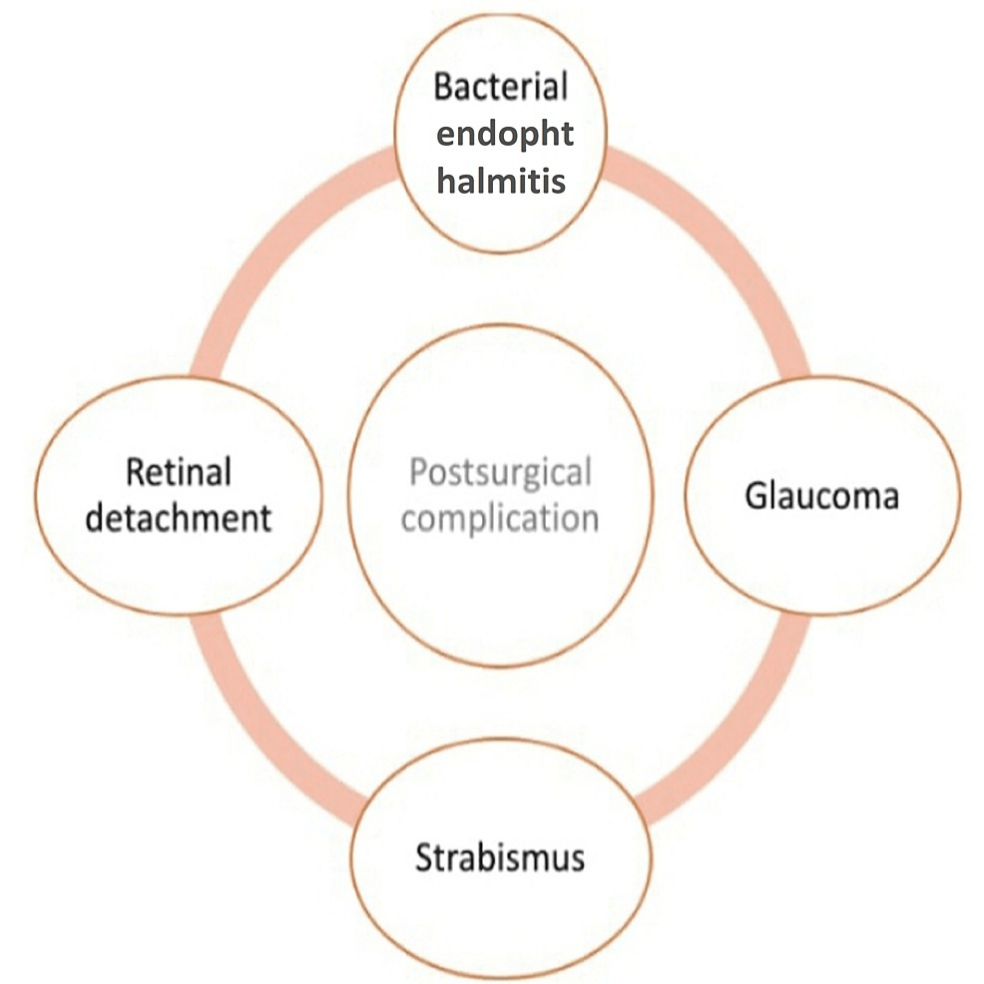
Postsurgical complications arising due to cataract surgery. The image is created by the authors of this study.

Postsurgical complications arising due to cataract surgery, including bacterial endophthalmitis, retinal detachment, glaucoma, and strabismus, are shown in Figure 2.

*Visual* *Axis* *Opacification*

In pediatric cataract surgery most commonly noted complication of surgery is visual axis opacification (VAO). The child's eyes are highly elastic and have enhanced immune response. VAO depends on the age at which the child is operated on. Younger children undergoing surgery have a lesser incidence of VAO. Opacification of the axis can be controlled by primary management of anterior vitrectomy and posterior capsule. Level of opacification depends on the type of IOL used and at which place IOL is implanted. Incidence of posterior capsule opacification can be reduced by placing the IOL in a lens bag. Sealed capsule irrigation devices used in cataract surgery reduced complications of visual axis opacification incidence [34].

*Secondary* *Glaucoma*

**Figure 3:**
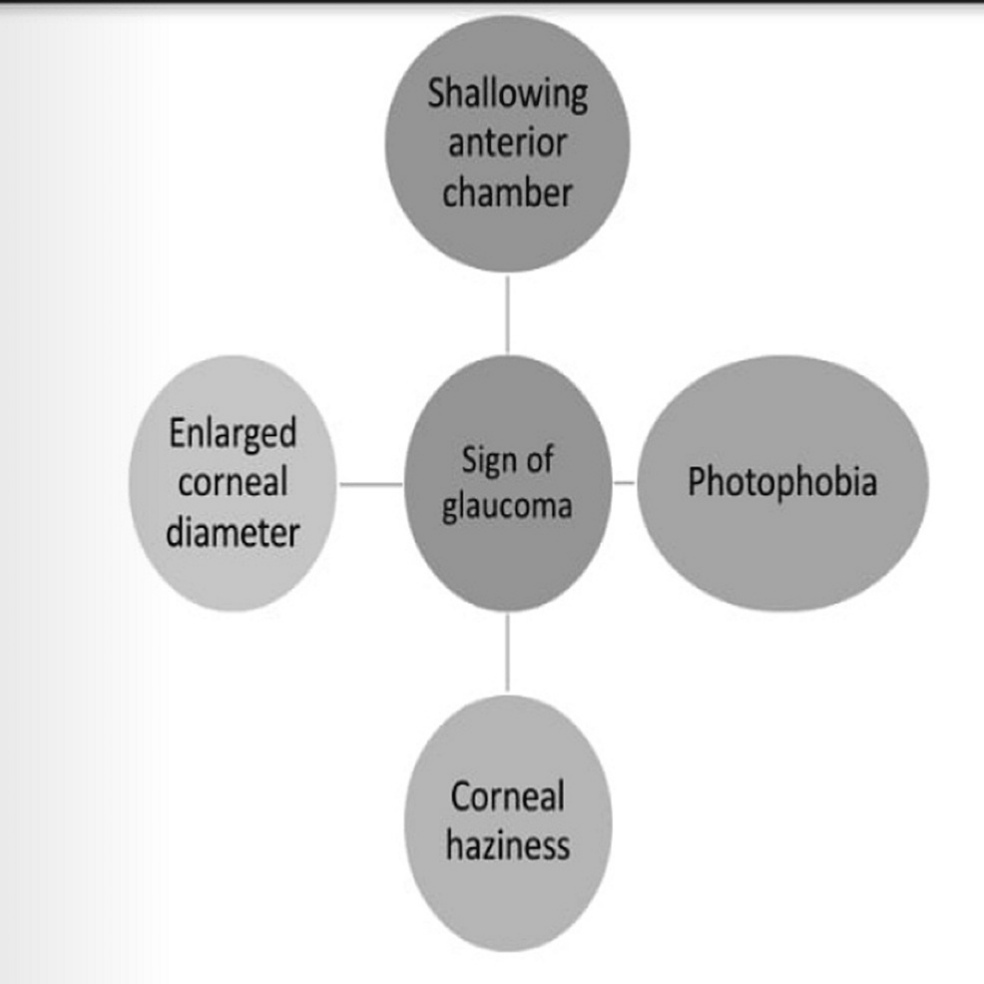
Presentation of glaucoma in children as complication of childhood cataract surgery. The image is created by the authors of this study.

The incidence of secondary glaucoma remains high despite outstanding surgery. According to many ophthalmologists, it occurs due to aphakia. Open-angle glaucoma is commonly reported in children. Factors responsible for glaucoma include ocular abnormalities, age at operation, type of IOL used, lens protein, the case with nuclear cataract, and small size of cornea [34]. Secondary glaucoma is the result of cataract surgery identified through the signs shown in Figure 3.

## Conclusions

Childhood blindness can be reduced by understanding different etiologies and clinical presentation of congenital cataracts. Early diagnosis of cataract in a child can be made with ultrasonography. Screening should be done in the maternity ward. Parental counseling for hereditary cataracts is important. In children, eye surgery should be done with precaution as the eye a is growing structure in the child. In mature cataract with nystagmus, if surgery is not performed it can lead to development of irreversible amblyopia. In all children, anterior and posterior capsulorhexis is done and IOL is implanted only in children more than one year of age with bilateral cataract. Lifelong follow-up is required to check for postoperative complications such as secondary glaucoma.
